# Compound Heterozygous Variants of the *CPAMD8* Gene Co-Segregating in Two Chinese Pedigrees With Pigment Dispersion Syndrome/Pigmentary Glaucoma

**DOI:** 10.3389/fgene.2022.845081

**Published:** 2022-07-25

**Authors:** Junkai Tan, Liuzhi Zeng, Yun Wang, Guo Liu, Longxiang Huang, Defu Chen, Xizhen Wang, Ning Fan, Yu He, Xuyang Liu

**Affiliations:** ^1^ Xiamen Eye Center, Xiamen University, Xiamen, China; ^2^ Department of Ophthalmology, Chengdu First People’s Hospital, Chengdu, China; ^3^ Shenzhen Eye Hospital, Shenzhen Eye Institute, Jinan University, Shenzhen, China; ^4^ Sichuan Provincial Key Laboratory for Human Disease Gene Study, Sichuan Provincial People’s Hospital, University of Electronic Science and Technology of China, Chengdu, China; ^5^ Department of Ophthalmology, The First Affiliated Hospital of Fujian Medical University, Fuzhou, China; ^6^ School of Ophthalmology and Optometry, The Eye Hospital, Wenzhou Medical University, Wenzhou, China; ^7^ Department of Ophthalmology, Shenzhen People’s Hospital, The 2nd Clinical Medical College, Jinan University, Shenzhen, China

**Keywords:** CPAMD8, pigmentary glaucoma, autosomal recessive inheritance, pedigree, compound heterozygous variant

## Abstract

The molecular mechanisms underlying the pathogenesis of pigment dispersion syndrome and pigmentary glaucoma remain unclear. In pedigree-based studies, familial aggregation and recurrences in relatives suggest a strong genetic basis for pigmentary glaucoma. In this study, we aimed to identify the genetic background of two Chinese pedigrees with pigmentary glaucoma. All members of these two pedigrees who enrolled in the study underwent a comprehensive ophthalmologic examination, and genomic DNA was extracted from peripheral venous blood samples. Whole-exome sequencing and candidate gene verifications were performed to identify the disease-causing variants; in addition, screening of the *CPAMD8* gene was performed on 38 patients of sporadic pigmentary glaucoma. Changes in the structure and function of abnormal proteins caused by gene variants were analyzed with a bioinformatics assessment. Pigmentary glaucoma was identified in a total of five patients from the two pedigrees, as were compound heterozygous variants of the *CPAMD8* gene. No signs of pigmentary glaucoma were found in carriers of monoallelic *CPAMD8* variant/variants. All four variants were inherited in an autosomal recessive mode. In addition to the 38 patients of sporadic pigmentary glaucoma, 13 variants of the *CPAMD8* gene were identified in 11 patients. This study reported a possible association between *CPAMD8* variants and pigment dispersion syndrome/pigmentary glaucoma.

## Introduction

Pigment dispersion syndrome (PDS) is a relatively rare ocular disorder that affects mostly males, especially in Caucasian countries (Niyadurupola and Broadway, 2008). PDS can lead to pigmentary glaucoma (PG), a type of open-angle glaucoma secondary to excessive deposition of pigment in the trabecular meshwork (TM). The deposited pigment blocks the conventional outflow pathway, leading to an increased resistance to aqueous humor outflow and elevated intraocular pressure (IOP). It is generally believed that the dispersed pigment originates from the shedding of the pigment epithelium located on the posterior surface of the iris (Scuderi et al., 2019). In a prospective study, the criteria for PDS were set as two of three signs: Krukenberg’s spindle, iris transillumination defects, and/or TM pigmentation; in contrast, PG was diagnosed when two of three signs were seen in PDS eyes: IOP greater than 21 mmHg, glaucomatous optic nerve damage, and/or glaucomatous visual field (VF) loss (Tandon et al., 2019). However, iris transillumination defects were not common in East Asian populations probably because these defects cannot be detected in darkly pigmented irides of East Asian patients *via* slit-lamp examination (Qing et al., 2009). To date, the molecular mechanisms underlying the pathogenesis of PDS and PG remain unclear. In pedigree-based studies, familial aggregation and recurrences in relatives suggest a strong genetic basis for PG (Siddiqui et al., 2003). In addition, variants of the premelanosome (*PMEL*) gene were associated with heritable patients of PDS/PG (Lahola-Chomiak et al., 2019), and variants of the pigment dispersion–associated genes in animals, such as *Dct*, *Tyrp1*, and *Lyst*, mimicked some clinical features of PDS and PG (Chang et al., 1999; Anderson et al., 2008; Trantow et al., 2009); however, no studies have found a causal relationship between any variants within their human orthologs and PG. Therefore, it is likely that there are other genes involved in the pathogenesis of PDS/PG. Posterior bowing of the iris is frequently present in PDS/PG, causing iridozonular contact, zonular rubbing, and pigment dispersion, which results in excessive pigment deposition in the anterior chamber angle tissues, especially the TM, as aqueous humor and dispersed pigment follow the normal aqueous humor dynamics through the outflow pathway (Lyons and Amram, 2019). Giardina et al. identified the haplotypes of the *LOXL1* gene in a cohort of patients with PDS/PG and validated the pathogenic effects of the *LOXL1* variant in an animal model. They also speculated that *LOXL1* variant–induced defects of the iris stroma are the reason for iris concavity, a phenotype often present in PDS/PG patients (Giardina et al., 2014). However, another study showed that there was no correlation between *LOXL1* and PDS/PG (Rao et al., 2008). Hence, the specific pathogenic factor and mechanism of PDS/PG remain poorly understood. Nevertheless, these studies indicate that genetic factors could be associated with an increased risk of developing PDS/PG.

The human *CPAMD8* gene (complement 3- and pregnancy zone protein–like, alpha-2-macroglobulin domain–containing 8; OMIM 608841) encodes a protein involved in the immune system (Nagase et al., 1999; Li et al., 2004; Wiggs, 2020). Variants of the *CPAMD8* gene have been recently identified as causative factors for anterior segment dysgenesis (ASD) (Cheong et al., 2016; Bonet-Fernandez et al., 2020; Ma et al., 2020), morgagnian cataract (Hollmann et al., 2017), and different subtypes of glaucoma, including POAG, PACG, and congenital glaucoma (Bonet-Fernandez et al., 2020; Siggs et al., 2020; Wiggs, 2020; Li X. et al., 2021b), indicating that *CPAMD8* variants may lead to primary and secondary glaucoma; different variants of the *CPAMD8* gene may also determine the presentation and severity of ocular phenotypes.

In the current study, compound heterozygous *CPAMD8* variants were identified in two pedigrees with PG. Moreover, autosomal recessive inheritance was found in the study of PG pedigrees. Our study reported a possible association between the biallelic *CPAMD8* gene variants and PDS/PG.

## Methods

### Human Subjects

Two Chinese pedigrees of PG and 38 sporadic PG patients were recruited at Shenzhen Eye Hospital and Chengdu First People’s Hospital, respectively. Members of both pedigrees and sporadic PG patients were systematically interviewed about their medical history and clinically examined by experienced glaucoma specialists; in addition, peripheral blood was collected for DNA analysis. This study was approved by the Medical Ethics Committees of Shenzhen Eye Hospital, Chengdu First People’s Hospital, and Xiamen Eye Center and was conducted according to the principles of the Declaration of Helsinki. Informed written consent was obtained from all participants prior to inclusion in the study.

### Clinical Examination

All members enrolled in the study underwent a comprehensive ophthalmologic examination, which included best corrected visual acuity, IOP measurement, slit-lamp biomicroscopy, gonioscopy, and fundus examination. The ocular biometric parameters of each participant were measured *via* optical biometry (IOL Master 700, Carl Zeiss, Germany). Glaucomatous optic neuropathy (GON) was evaluated using slit-lamp examination with a concave preset lens (Hruby lens), fundus photography, and spectral domain optical coherence tomography (SD-OCT, Carl Zeiss). VF was examined using the Humphrey VF Analyzer (Carl Zeiss). The anterior chamber angle structure was recorded *via* ultrasound biomicroscopy (UBM, Tianjin Suowei Electronic Technology Co., Ltd., China) and gonioscopic photography.

The PDS/PG was diagnosed according to the criteria previously reported (Tandon et al., 2019) with minor modifications. In brief, for PDS, subjects should have two signs in at least one eye as follows: Krukenberg’s spindle and heavily pigmented TM (Grade 3 or higher). For PG, subjects should have PDS plus at least two of the three signs in at least one eye as follows: IOP > 21 mmHg, GON (C/D ratio > 0.5), and glaucomatous VF defect. The degree of TM pigmentation was graded from 0 to 4 according to severity (0 = no pigment; 1 = light pigment; 2 = moderate pigment; 3 = heavy and non-confluent pigment; and 4 = heavy and confluent pigment) (Tandon et al., 2019). The severity of Krukenberg’s spindle was also graded from 0 to 4 (0 = none; 1 = few flecks; 2 = subtle spindle; 3 = dense spindle; 4 = diffuse pigment) (Tandon et al., 2019).

### Whole-Exome Sequencing and Candidate Gene Validation

Genomic DNA of all individuals was extracted from peripheral venous blood samples using the Qiamp Blood DNA Mini Kit (QIAGEN GmbH, Hilden, Germany) according to the manufacturer’s instructions. DNA integrity was evaluated *via* 1% agarose gel electrophoresis. Whole-exome sequencing (WES) was performed on three patients in pedigree 1 and the proband in pedigree 2 by Shanghai Genesky Biotechnologies, Inc., and Shanghai WeHealth BioMedical Technology, Co., Ltd, respectively, according to previously described methods (Bao et al., 2019). Exons and adjacent splicing regions (about 20 bp), as well as the full length of the mitochondrial genome, were captured and enriched by SeqCap EZ Exome Probes v3.0 (Roche, Switzerland) hybridization. After the quality control steps, the enriched genes were sequenced using an Illumina HiSeq X Ten platform (San Diego, CA).

The original WES data, excluding unqualified reads according to the quality control standard, were compared with the human reference genome sequence from the University of California Santa Cruz Genome Browser Database using the BWA software (hg19 version). The single-nucleotide variation and insertion and deletion (indel) variants were found using HaplotypeCaller of the genome analysis toolkit. The identified variants were classified *in silico* and filtered against the genome aggregation database (gnomAD, version 3.1, https://gnomad.broadinstitute.org/). Variants were filtered for minor allele frequency (MAF) ≤ 1% (0.01) in gnomAD. Professional databases and bioinformation prediction software, including SnapGene (version 4.3, www.snapgene.com), Ensembl (https://asia.ensembl.org/index.html), DUET (http://biosig.unimelb.edu.au/duet/stability), and VarSite (https://www.ebi.ac.uk/thornton-srv/databases/cgi-bin/VarSite/GetPage.pl?home=TRUE), were used for further annotation and screening of variants of interest, such as missense, synonymous, and noncanonical splicing variants, according to reference sequences NM_015692.5 and NP_056507.3. SnapGene was used to align the protein sequences among different species. The possible functional impact of an amino acid change was predicted using PolyPhen-2, SIFT, CADD, DUET, and VarSite. The first three prediction data (PolyPhen-2, SIFT, and CADD) were acquired from the Ensembl website for uniforming their versions used in the study. The XHMM and CLAMMS algorithms were employed to analyze the copy number variation of the probe coverage area.

The variant-filtering steps were summarized as follows: according to the autosomal recessive inheritance or compound heterozygous inheritance model, the exome sequencing data of patients from family 1 (II:1, II:4, and II:5) and family 2 (II:3) were screened. Homozygous variants and two or more heterozygous variants of the same gene were obtained after filtration. Genes fully or partially related to clinical phenotype were taken into consideration. Glaucoma-associated genes, including *MYOC*, *CYP1B1*, *FOXC1*, *LRP2*, *PITX2*, *PAX6*, *LTBP2*, *TEK*, *ANGPT1*, and *PMEL*, were given special attention. Information on candidate genes and variants from both families were obtained through literature and human gene mutation database (HGMD) search using the American College of Medical Genetics and Genomics (ACMG)/AMP (PMID) criteria (Richards et al., 2015).

### Variant Analyses

Direct Sanger sequencing was used to determine the co-segregation of identified variants with the clinical phenotype in pedigrees. Candidate variants identified through the aforementioned variant-filtering steps were validated by Sanger sequencing in all members of the two pedigrees. The primer flanking regions of candidate variants of the *CPAMD8* gene (Gene ID 27151) were designed using Primer Premier 5 (Table 1) and synthesized by BGI (Guangzhou, China). Polymerase chain reaction (PCR) was performed using the MyCycler thermal cycler (Bio-Rad, Hercules, CA) in a 25-µL reaction system, which contained 0.1-µg genomic DNA, 40-μmol/L forward and reverse primers, 3-mmol/L magnesium chloride, and 2 × Taq Master Mix (SinoBio, Shanghai, China). The PCR conditions used were as follows: 5 min at 95°C for initial denaturation; 35 cycles of denaturation at 95°C for 30°s for melting; corresponding annealing temperature (TM value) in Table 1 lasting for 30°s; 30°s at 72°C for extension; and a final additional extension step of 10 min at 72°C. Before sequencing, 1% agarose gel electrophoresis was used to purify the target PCR fragments using the QIAquick Gel Extraction Kit (QIAGEN, Shanghai, China). Sanger sequencing was performed on an ABI 377XL automated DNA sequencer (Applied Biosystems, Foster City, CA). Sequence data were compared in a pairwise manner with the related human genome database.

**TABLE 1:** Primers used in polymerase chain reaction (PCR) for the amplification of the *CPAMD8* gene.

Variants	Primer sequence (forward/reverse)	Product size (bp)	TM value (°C)
c.520C>T, p.R174W	5′-ACA​GTC​ACC​CCC​AAG​TTA​CC-3′	266	56
	5′-GTG​TTT​TTC​CTC​CCT​CCA​GA-3′		
c.1015G>A, p.V339M	5′-CAG​AGG​GTC​CAA​ATC​CAC​AG-3′	385	55
	5′-ATG​CCC​AAG​AGA​GAG​GAC​TG-3′		
c.1931A>G, p.Y644C	5′-TCC​AAT​TCT​GTT​TCC​AAC​CCA-3′	510	60
	5′-GGC​TGG​TCT​CGA​ACT​CCT​TT-3′		
c.3238G>A, p.G1080S	5′-CAG​CTC​TTG​GGC​TTC​CTC​AA-3′	520	59
	5′-CAC​AAA​GCT​GGT​GTC​ACT​GC-3′		

### Screening of the CPAMP8 Gene in Sporadic Pigmentary Glaucoma Patients

The *CPAMP8* gene was screened in 38 sporadic PG patients *via* WES as described above. Variants were filtered with gnomAD (East Asian population); variants with MAF ≤ 0.01 were considered variants or functional SNPs (non-synonymous SNPs).

## Results

### Clinical Features of Pigmentary Glaucoma Patients

Five patients from two pedigrees who carried biallelic *CPAMD8* compound heterozygous variants presented with typical clinical phenotypes of PDS/PG, including Krukenberg’s spindle on the posterior surface of the cornea, heavily pigmented TM, elevated IOP, and GON. The clinical data of the patients in the two PG pedigrees with biallelic *CPAMD8* variants were summarized in Table 2.

**TABLE 2:** Clinical data of patients in two pigmentary glaucoma (PG) pedigrees.

Pedigree	Patient	Diagnosis	Gender	Changes of nucleotide and amino acid	Age at last exam (years old)	BCVA		Last IOP (mmHg)		Krukenberg’s spindle^a^		TM pigmentation^b^		C/D ratio		Visual field		RNFL (μm)	
						OD	OS	OD	OS	OD	OS	OD	OS	OD	OS	OD	OS	OD	OS
1	II:1	PG OU	M	c.520C>T, p.R174W/c.1015G>A, p.V339M	40	1.0	HM	15	19	4	4	4	4	0.8	0.9	NS	TI	58	57
	II:4	PDS OD; PG OS	F	c.520C>T, p.R174W/c.1015G>A, p.V339M	39	1.0	1.0	22	23	2	2	3	3–4	0.6	0.6	BSE	NS	101	89
	II:5	PG OU	M	c.520C>T, p.R174W/c.1015G>A, p.V339M	37	1.0	0.3	20	19	4	4	4	4	0.8	0.8	NS	PS	76	57
2	II:2	PG OU	F	c.1015G>A, p.V339M/c.1931A>G, p.Y644C/c.3238G>A, p.G1080S	48	NLP	0.3	36	15	NA	4	NA	4	NA	0.9	NA	TI	NA	48
	II:3	PG OU	F	c.1015G>A, p.V339M/c.1931A>G, p.Y644C/c.3238G>A, p.G1080S	46	0.3	0.6	14	14	3	3	4	4	0.9	0.8	TI	TI	45	54

Abbreviations: BCVA, best corrected visual acuity; BSE, blind spot enlargement; C/D, cup-to-disc; F, female; HM, hand move; IOP, intraocular pressure; M, male; N, normal; NA, not available; NLP, no light perception; NS, nasal step; OD, right eye; OS, left eye; OU, both eyes; PDS, pigment dispersion syndrome; PG, pigmentary glaucoma; PS, paracentral scotoma; RNFL, retinal nerve fiber layer; TI, temporal island; TM, trabecular meshwork.

a Grade of Krukenberg’s spindle: 0 is defined as none; 1, few flecks; 2, subtle spindle; 3, dense spindle; 4, diffuse pigment (Tandon et al., 2019).

b Grade of TM, pigmentation: 0 is defined as no pigment; 1, light pigment, 2, moderate pigment; 3, heavy and non-confluent pigment; 4, heavy and confluent pigment (Tandon et al., 2019).

**FIGURE 1:**
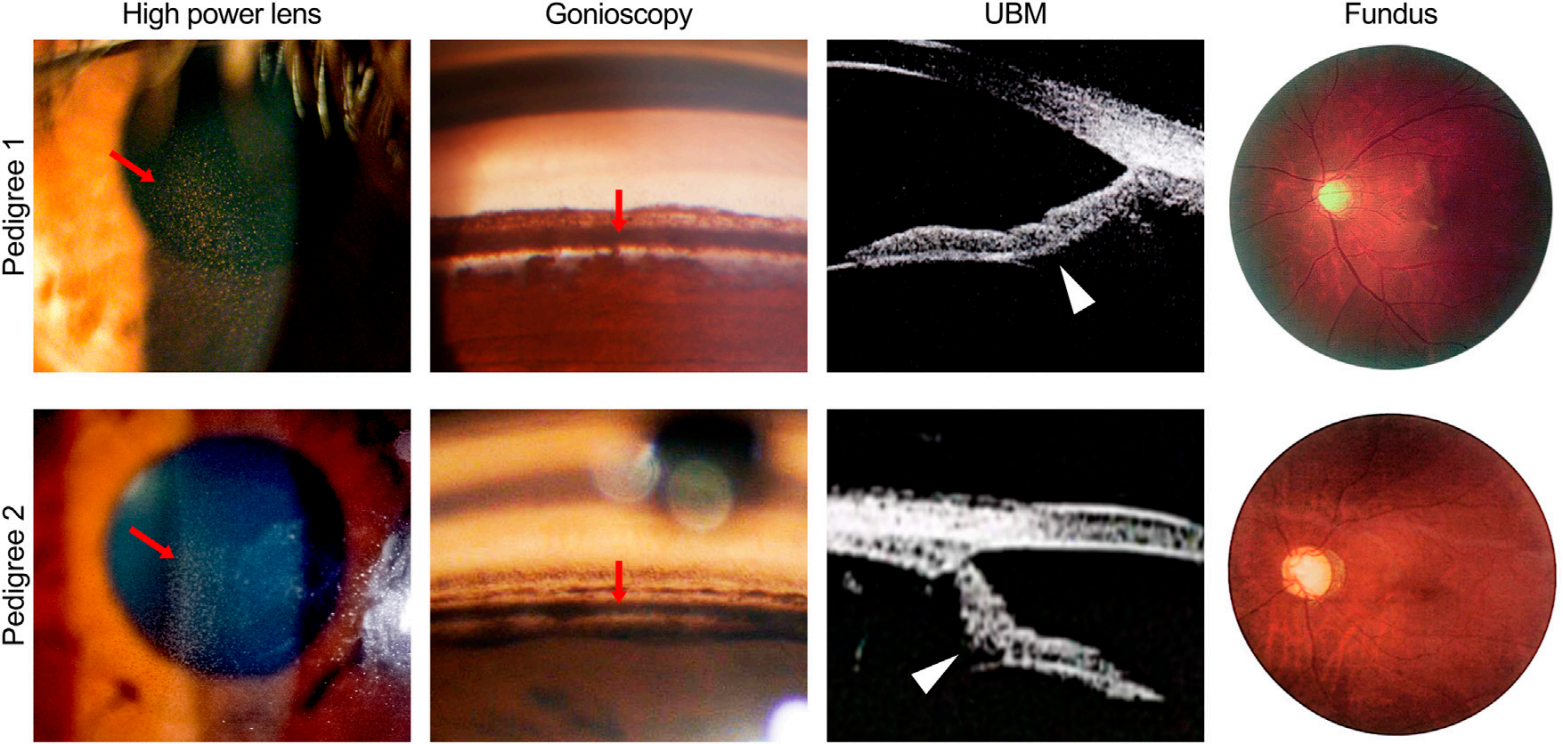
Ocular phenotypes of patients in two pigmentary glaucoma (PG) pedigrees associated with *CPAMD8* variants. Clinical photography including slit-lamp biomicroscopy, gonioscopy, ultrasound biomicroscopy, and fundus photography. Representative signs of PG, such as Krukenberg’s spindle, TM pigmentation, and reverse pupillary block, were indicated by red arrows and white arrowheads, respectively. In pedigree 1, representative images were acquired from the right eye of patient II:5. In pedigree 2, representative images were acquired from the left eye of patient II:2 (slit-lamp biomicroscopy and fundus photography) and the right eye of patient II:3 (gonioscopy and ultrasound biomicroscopy).

A relatively mild phenotype was observed in patient II:4 from pedigree 1, including subtle Krukenberg’s spindle and moderate pigmentation of the TM in both eyes, with elevated IOP, VF damage, and inferotemporal retinal nerve fiber layer (RNFL) defect in the left eye (Table 2). The patient’s elder and younger brothers, the other two patients in pedigree 1 (II:1 and II:5), presented with more significant glaucomatous manifestations, including increased IOP and GON. Diffuse Krukenberg’s spindle and grade 4 TM pigmentation were observed in both eyes. An enlarged C/D ratio (0.8–0.9), VF defects, and thinning RNFL confirmed the diagnosis of GON. UBM photographs of patient II:1 and II:5 (pedigree 1) revealed that the iris was bowed backward bilaterally, creating a reverse pupillary block and leading to a possible contact between the posterior surface of the iris and the zonules or anterior lens capsule (Figure 1 and Table 2).

The proband of pedigree 2 (II:3), a 46-year-old woman, underwent trabeculectomy in the right eye 2 years ago and presented with high myopia, poor corrected visual acuity, and controlled IOP with IOP-lowering medications in both eyes. Krukenberg’s spindle, lens opacity, and heavily pigmented TM were seen in both eyes. Bilateral typical GON with C/D ratio close to 1.0 was also observed (Figure 1 and Table 2).

The proband’s sister (II:2) from pedigree 2 had undergone glaucoma surgeries three or four times and presented with poor visual acuity and elevated IOP in both eyes. Due to the uncontrolled IOP and corneal endothelial decompensation in the right eye, the KP, TM, and optic disc could not be observed in detail. Krukenberg’s spindle, homogeneously and densely pigmented TM, and GON were detected in the left eye. UBM examination revealed a concave iris in both eyes of II:2 and II:3 in pedigree 2 (Figure 1 and Table 2).

The IOP presented in Table 2 were measured at the last follow-up visit of the patients, prior to which both II:1, II:5 in Family 1 and II:2, II:3 in Family 2 had undergone a single or multiple anti-glaucoma surgeries; five patients are currently treated with at least one anti-glaucoma drug. All the affected individuals, but not healthy individuals, in this study presented with elevated IOP before and during the treatments.

Elevated IOP, Krukenberg’s spindle, homogeneously and densely pigmented TM, and GON were also seen in 38 sporadic PG patients (data not shown). As expected, iris transillumination was not observed in patients enrolled in this study.

Carriers of the monoallelic *CPAMD8* variant/variants were asymptomatic (Supplementary Table S1), except for the mother of two affected daughters in pedigree 2, who had a long history of inflammatory keratitis and presented with leucoma and normal IOP in both eyes. She denied a history of elevated IOP.

### Variant Identification of *CPAMD8* in Two Pigmentary Glaucoma Pedigrees

**FIGURE 2:**
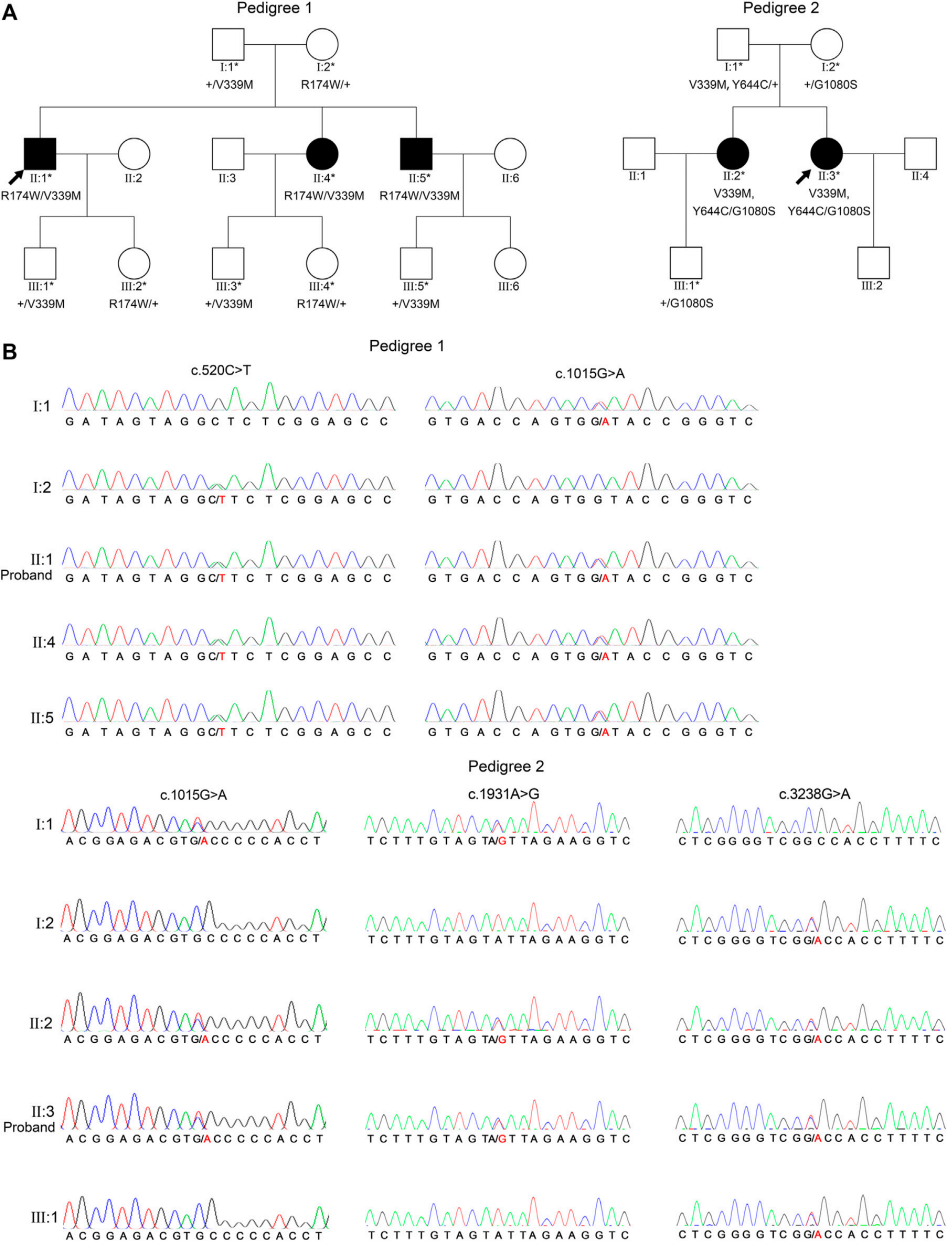
Two PG pedigrees with *CPAMD8* variants and Sanger sequencing results of the *CPAMD8* gene. **(A)** The two PG pedigrees described from our research. Round symbols indicate female individuals; square symbols indicate male individuals; black symbols indicate PG patients; arrow marks indicate the proband; asterisks represent subjects involved in genetic research. The plus signs denote reference allele. **(B)** Sanger sequencing verification and segregation analysis of biallelic *CPAMD8* variants in two PG pedigrees. Variants are denoted in red font. Two different compound heterozygous variants of *CPAMD8* were identified in two PG pedigrees. Abbreviations: PG, pigmentary glaucoma.

Common genetic characteristics were found in both three-generation pedigrees as follows: no history of consanguineous marriage; all patients were first filial generation; parents and second filial generation were not diagnosed with either PDS or PG; and segregation was consistent with autosomal recessive inheritance. WES and Sanger sequencing were used to investigate the underlying genetic alterations in the two pedigrees as described below, using pedigree 2 as an example (Figure 2A). The original sequencing data was 11,820.95 MB, and the average sequencing depth of the target region was 142.71X, with a 98.15% coverage of average sequencing depth of up to ×30. No pathogenic or possible pathogenic variation of mitochondrial genome and no copy number variation fully or partially related to the clinical phenotype of the patients were detected. After performing the variant-filtering steps according to the autosomal recessive inheritance or compound heterozygous inheritance model, a total of 176 homozygous variants of 67 genes (Supplementary Table S2) or two or more heterozygous variants of the same genes were shared by all affected individuals (II:1, II:4, and II 5) of family 1; on the contrary, a total of 230 variants of 100 genes (Supplementary Table S3) using similar filtering strategy were identified in the proband from family 2 (II:3). Information of all candidate genes in both families was obtained through literature and HGMD search, in which only *CPAMD8* was previously reported in relation to glaucoma (Bonet-Fernandez et al., 2020; Siggs et al., 2020; Wiggs, 2020; Li X. et al., 2021b). Furthermore, Sanger sequencing verification of *CPAMD8* was carried out with all family members in both pedigrees. It was confirmed that compound heterozygous variants (c.520C>T, p.R174W and c.1015G>A, p.V339M in pedigree 1; c.1015G>A, p.V339M; c.1931A>G, p.Y644C and c.3238G>A, p.G1080S in pedigree 2) in *CPAMD8* were co-segregated in two pedigrees with PDS/PG patients (patient II:1, II:4, and II:5 from pedigree 1; patient II:2 and II:3 from pedigree 2; Figure 2A). All variants in this study were rare, with one (p.G1080S) unavailable in gnomAD (East Asian), at a frequency consistent with that of rare recessive disease (Table 3).

**TABLE 3:** Summary of PG pedigrees with *CPAMD8* Variants.

Pedigree	Position	Name	Exon	Changes of nucleotide and amino acid	Status	Variant type	PolyPhen-2	CADD	SIFT	DUET-protein stability	gnomAD_EA	ACMG/AMP variant classification
1	chr19:17119354	*CPAMD8*	7	c.520C>T, p.R174W	CH	Missense	0.999, D	23, B	0.01, D	-0.216 kcal/mol, D	0.00019	VUS^a^
	chr19:17108001	*CPAMD8*	11	c.1015G>A, p.V339M	CH	Missense	0.999, D	23, B	0.02, D	-0.822 kcal/mol, D	0.00347	VUS^b^
2	chr19:17108001	*CPAMD8*	11	c.1015G>A, p.V339M	CH	Missense	0.999, D	23, B	0.02, D	-0.822 kcal/mol, D	0.00347	VUS^b^
	chr19:17086046	*CPAMD8*	17	c.1931A>G, p.Y644C	CH	Missense	0.921, D	22, B	0.01, D	-1.977 kcal/mol, D	0.00019	VUS^a^
	chr19:17038951	*CPAMD8*	25	c.3238G>A, p.G1080S	CH	Missense	0.883, D	23, B	0.17, T	-1.91 kcal/mol, D	NA	VUS^a^

Abbreviations: CH; compound heterozygous; D, damaging, deleterious, or destabilizing; EA, East Asian; NA, not available; T, tolerated; VUS, variants of uncertain significance.

a Evidence of pathogenicity (PM2 + PP1 + PP3 + PP4).

b Evidence of pathogenicity (PP1 + PP3 + PP4).

### Bioinformatics Analyses of the *CPAMD8* Variants in Two Pigmentary Glaucoma Pedigrees

In pedigree 1, compound heterozygous variants p.R174W (rs201333165) and p.V339M (rs369985652) in the *CPAMD8* gene were identified *via* WES. The genotype-phenotype co-segregation was verified *via* Sanger sequencing in the whole pedigree. Variant p.R174W of *CPAMD8* was inherited from the mother, whereas the other variant, p.V339M, was inherited from the father. The mode of inheritance was in accordance with compound heterozygous variation under a recessive inheritance pattern (Figure 2).

**FIGURE 3:**
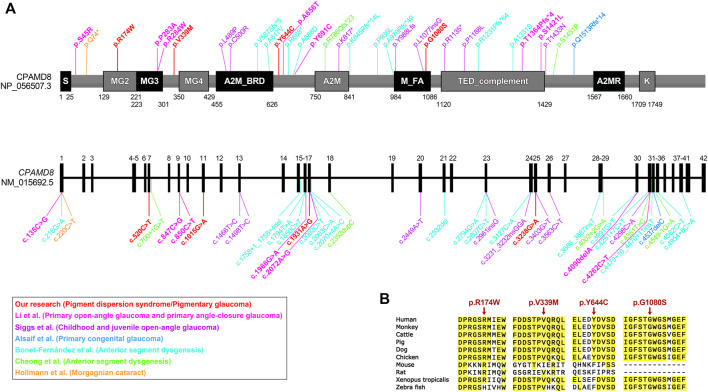
Bioinformatics analyses of *CPAMD8* variants in two PG pedigrees. **(A)** Schematic representations showing variant sites on the sequence of the *CPAMD8* gene and CPAMD8 protein identified in our study and other studies of the association between *CPAMD8* gene and inherited ocular disorders (Cheong et al., 2016; Hollmann et al., 2017; Alsaif et al., 2019; Bonet-Fernandez et al., 2020; Siggs et al., 2020; Li X. et al., 2021b). Each study was plotted in a distinct color. Domains were indicated according to the Pfam database (https://pfam.xfam.org/). Abbreviations: S, signal peptide; A2M, α-2-macroglobulin domain; A2M_BRD, α-2-macroglobulin bait region domain; A2MR, α-macroglobulin receptor binding domain; MG, macroglobulin domain of α-2-macroglobulin; M_FA, farnesoic acid O-methyl transferase domain; TED, α-macroglobulin thioester domain; K, kazal-2 serine protease inhibitor domain. **(B)** Sequence alignments of the CPAMD8 protein among different species. Four missense variants were highly conserved in various species. The positions of the variant amino acids are indicated by red arrows.

Two missense variants found in the *CPAMD8* gene of the subject were classified as “variants of uncertain significance” according to the ACMG guidelines. Three online predictive software, PolyPhen-2, SIFT, and DUET, classified these two variants as damaging, destabilizing, or deleterious (Table 3). The macroglobulin domain 2 (MG2) (Figure 3A) is a domain of α-2-macroglobulin (A2M) in eukaryotes (Janssen et al., 2005). A2Ms, among the core domains of *CPAMD8*, are plasma proteins that trap and inhibit a broad range of proteases and are major components of the eukaryotic innate immune system (Wong and Dessen, 2014). The arginine (Arg) residue at position 174 is highly conserved (Figure 3B; conservation = 0.9 from 37 aligned protein sequences from different organisms). The change from an Arg to a tryptophan side chain was highly unfavored in terms of conserved amino acid properties, which might result in a change in protein function.

In contrast to Arg at position 174, the valine residue at position 339 is relatively less conserved (Figure 3B; conservation = 0.7 from 43 aligned protein sequences from different organisms). This may not result in an obvious change in protein function.

In pedigree 2, three variants, p.V339M, p.Y644C, and p.G1080S, in the *CPAMD8* gene were identified *via* WES. Two variants, p.V339M and p.Y644C, were inherited from their father, whereas another variant, p.G1080S, was inherited from their mother. These variants were co-segregating with the clinical phenotype verified *via* Sanger sequencing and segregation analysis and were accordant with compound heterozygous variation under a recessive inheritance pattern (Figure 2).

Three missense variants found in the *CPAMD8* gene of the subject were classified as “variants of uncertain significance” according to the ACMG/AMP guidelines for variant classification. Variant p.V339M was presented in both pedigrees. Another two variants (p.Y644C and p.G1080S) were identified as highly conservative in different species (Figure 3B) and might result in instability of the CPAMD8 protein, as predicted by DUET (Table 3). The p.Y644C and p.G1080S variants were predicted to be damaging or deleterious by PolyPhen-2. SIFT predicted that the p.Y644C variant was deleterious and the p.G1080S variant was tolerable (Table 3). The heterozygous variant c.1931A>G of exon 17 in *CPAMD8* would result in the substitution of tyrosine (Tyr), a neutral amino acid at position 644, by hydrophobic cysteine. The Tyr residue at position 644 is very highly conserved (Figure 3B; conservation = 0.9 from 43 aligned protein sequences from different organisms), rendering it significant for protein function.

The heterozygous variant c.3238G>A of exon 25 in *CPAMD8* would result in the substitution of glycine (Gly) at position 1080 by serine, which is located at the end of the methyltransf-FA (farnesoic acid O-methyl transferase) domain (Figure 3A), a well-known key protein domain of the enzyme that catalyzes the formation of methyl farnesoate from farnesoic acid in the biosynthetic pathway of juvenile hormone (Li J. et al., 2021a). The Gly residue at position 1080 is fairly well conserved (Figure 3B; conservation = 0.8 from 47 aligned protein sequences), which means it is significant for protein function.

### Screening of the *CPAMD8* Gene in Sporadic Pigmentary Glaucoma Patients

By screening the *CPAMD8* gene on 38 sporadic PG patients (Table 4), 13 variants (c.1015G>A, c.503T>C, c.5242C>G, c.1173_1174del, c.92+1G>T, c.3786+1G>A, c.2117G>A, c.215C>T, c.959C>T, c.979G>T, c.2009G>A c.5342C>G, and c.4685+1G>A) of the *CPAMD8* gene with MAF ≤0.01 were identified in 11 sporadic PG patients. Among them, one variant of *CPAMD8* (c.1015G>A, p.V339M) was identified in pedigree 1 and pedigree 2 as well as one sporadic patient (patient 1) of PG, which was not reported previously. In addition, one sporadic PG patient (patient 2) carried three heterozygous variants of *CPAMD8*.

**TABLE 4:** Summary of sporadic PG patients with *CPAMD8* variants.

Patient number	Position	Name	Exon	Changes of nucleotide and amino acid	Variant type	PolyPhen-2	CADD	SIFT	DUET-protein stability	gnomAD_EA
1	chr19:17108001	*CPAMD8*	11	c.1015G>A, p.V339M	Missense	0.999, D	23.0, B	0.02, D	-0.822 kcal/mol, D	0.00347
	chr19:17120114	*CPAMD8*	6	c.503T>C, p.L168P	Missense	0.966, D	22.6, D	0.310, T	-1.036 kcal/mol, D	NA
2	chr19:17007300	*CPAMD8*	40	c.5242C>G, p.L1748V	Missense	0.103, B	16.99, B	0.189, T	-1.648 kcal/mol, D	NA
	chr19:17104318_17104319	*CPAMD8*	12	c.1173_1174del, p.D391Efs*6	Frameshift	NA	NA	NA	NA	0.0001926
	chr19:17137360	*CPAMD8*	1	c.92+1G>T	Splicing	NA	33.0, D	NA	NA	NA
3	chr19:17025466	*CPAMD8*	28	c.3786+1G>A	Splicing	NA	33.0, D	NA	NA	NA
4	chr19:17081797	*CPAMD8*	18	c.2117G>A:p.R706Q	Missense	0.909, D	24, B	0.01, D	-0.239 kcal/mol, D	NA
5	chr19:17132869	*CPAMD8*	2	c.215C>T, p.P72L	Missense	0.01, B	11, B	0.05, D	-0.684 kcal/mol, D	0.01079
6	chr19:17108057	*CPAMD8*	11	c.959C>T, p.A320V	Missense	0.496, D	14, B	0.03, D	-0.192 kcal/mol, D	0.003277
7	chr19:17108037	*CPAMD8*	11	c.979G>T:p.G327W	Missense	1.000, D	25.10, D	0.001,D	-1.52 kcal/mol, D	NA
8	chr19:17085968	*CPAMD8*	17	c.2009G>A, p.R670Q	Missense	0.954, D	14, B	0.1, B	-0.22 kcal/mol, D	NA
9	chr19: 17007071	*CPAMD8*	41	c.5342C>G, p.P1781R	Missense	0, B	3, B	0.15, T	NA	0.00987
10	chr19:17108057	*CPAMD8*	11	c.959C>T, p.A320V	Missense	0.496, D	14, B	0.03, D	-0.192 kcal/mol, D	0.003277
11	chr19:17013458	*CPAMD8*	35	c.4685+1G>A	Splicing	NA	34.0, D	NA	NA	NA

Abbreviations: 1,000g2015aug_all, frequency of variation in 1,000 Genomes Project database (all population); 1,000g2015aug_eas; frequency of variation in 1,000 Genomes Project database (East Asian population); NA, not available; the variants from patient 1 (c.503T>C, p.L168P), 2 (c.5242C>G, p.L1748V and c.92+1G>T), 3, 4, 7, 8, and 11 were not available in the Ensembl website, and their prediction data including PolyPhen-2, SIFT, and CADD were acquired from their own websites or VarSite website; PolyPhen-2, (http://genetics.bwh.harvard.edu/pph2/); CADD, (https://cadd.gs.washington.edu/); SIFT, (http://provean.jcvi.org/); VarSite, (https://www.ebi.ac.uk/thornton-srv/databases/cgi-bin/VarSite/GetPage.pl?home=TRUE).

## Discussion

In this study, compound heterozygous variants of the *CPAMD8* gene were identified in five patients from two unrelated Chinese PG pedigrees *via* filtering of WES data and Sanger sequencing verification. These compound heterozygous variants of the *CPAMD8* gene have not been previously reported in literatures. All variants appear to be damaging, destabilizing, or deleterious based on bioinformatics analysis. Through WES and filtering against several publicly accessible variation databases and eliminating all previously reported variants, other glaucoma-associated genes, including *MYOC*, *CYP1B1*, *FOXC1*, *LRP2*, *PITX2*, *PAX6*, *LTBP2*, *TEK*, *ANGPT1*, and *PMEL*, were excluded in these two pedigrees. Of the 13 variants of *CPAMD8* identified in 38 sporadic PG patients@ with MAF ≤ 0.01, compound heterozygous variants were likely present in two of them (Table 4), and their parents were asymptomatic. These results indicate that the *CPAMD8* gene variant is potentially associated with PDS/PG.

In previous studies, PDS was thought to be an autosomal dominant inherited disease (Scheie and Cameron, 1981; Mandelkorn et al., 1983; Tandon et al., 2019); meanwhile, there was only one autosomal recessive inheritance pattern for PG in a four-generation family reported (Stankovic., 1961; Lascaratos et al., 2013). Andersen et al. (Andersen et al., 1997) used microsatellite repeat markers distributed throughout the human genome to perform a genome screen of subjects from four pedigrees with PDS. They found that the responsible gene is located in a 10-centimorgan interval between markers D7S2462 and D7S2423 of chromosome 7. The two pedigrees in this study presented an autosomal recessive inheritance pattern. No PDS/PG phenotypes were found in the carriers of monoallelic *CPAMD8* variant/variants from these two pedigrees. For pedigree 1, we have followed up the pedigree for 10 years and never found other pedigree members presenting with any phenotype of PDS/PG or glaucoma. In pedigree 2, patient I:1 with monoallelic *CPAMD8* variants (c.1015G>A, p.V339M; c.1931A>G, p.Y644C) had no previous history of ocular abnormalities, and examinations on his eyes were unremarkable. Therefore, our data meet the necessary criteria indicating that *CPAMD8* is involved in the inheritance of PG in a rare autosomal recessive pattern.

It was recently suggested that *CPAMD8* variants may be involved in the development of ASD (Cheong et al., 2016; Bonet-Fernandez et al., 2020; Ma et al., 2020), morgagnian cataract (Hollmann et al., 2017), and different subtypes of glaucoma, including POAG, PACG, and congenital glaucoma (Bonet-Fernandez et al., 2020; Siggs et al., 2020; Li X. et al., 2021b). However, neither ASD nor morgagnian cataract was present in our study of PG patients with *CPAMD8* missense variants, although these ocular disorders share similar background of changed *CPAMD8*. Li et al. performed a cohort study of the association between *CPAMD8* variants and POAG/PACG and showed that biallelic truncation variants were more frequently associated with ASD, which usually presented with more severe phenotype, including iridocorneal adhesion and iris and ciliary body hypoplasia, whereas biallelic missense variants were more common in POAG/PACG (Li X. et al., 2021b), suggesting that the functional damages of CPAMD8 protein could be different for different variants/alterations in the *CPAMD8* gene. The type of variants/alterations may determine the presentation and severity of the ocular phenotypes. In agreement with the previous report, all alterations identified in our study of PDS/PG with milder phenotypes than ASD were missense variants. We also found patient II:4 (female) of pedigree 1 who had later-onset and milder PG phenotypes (PDS in the right eye and PG in the left eye) than her elder and younger affected brothers, indicating that the clinical phenotypes of genetic disorders were possibly influenced by other factors, such as gender, epigenetic changes, and environmental exposure (Yan et al., 2010; Sarkar et al., 2014; Choi et al., 2020).

Variants of pigment dispersion–associated genes in animals, such as *Dct*, *Tyrp1*, and *Lyst*, mimicked some clinical features of PDS and PG (Chang et al., 1999; Anderson et al., 2008; Trantow et al., 2009); however, no studies have found a causal relationship between any variants within their human orthologs and PG. It is likely that there are non-pigment–related genes involved in the pathogenesis of PDS/PG. Iris concavity is often present in PDS/PG patients and leads to iridozonular chafing and pigment release (Lyons and Amram, 2019). Potential mechanisms for iridozonular chafing remain unclear. In a cohort study of PDS/PG patients, the *LOXL1* variant was identified and further validated to be pathogenic in an animal model. It was speculated that this variant was responsible for iris stromal defects in PDS/PG patients (Giardina et al., 2014). This study provided a possible interpretation for close contact of iris epithelium and zonule, that stromal defects appear to be present in abnormal iris. However, another study showed that there was no correlation between *LOXL1* and PDS/PG (Rao et al., 2008). An animal study examined the eyes of old DBA/2J mice (2.5–18 months), characterized its ocular pigment abnormalities, and speculated that the loss of pigment cells in the anterior chamber was at least in part caused by iris stromal atrophy with aging (Schraermeyer et al., 2009). This finding indicates that normal iris stroma may be necessary for the stability of pigment epithelium. In the anterior segment of the eye, *CPAMD8* has been demonstrated to be expressed in neural crest–derived tissues, including the cornea, iris, ciliary body, and lens (Wiggs 2020). Siggs et al. found iris stromal hypoplasia in childhood and juvenile POAG patients with biallelic *CPAMD8* variants (Siggs et al., 2020), which implied a possible role of *CPAMD8* in iris stromal defects. Furthermore, *CPAMD8* belongs to the α-2-macroglobulin/complement 3 protein family, whose members are involved in innate and acquired immune responses (Wiggs 2020). Wong et al. found that in the iris of patients with POAG or PCAG, the expression of IL-2 and IFN-γ (Th1 cytokine) increased, IL-6 (Th2 cytokine) decreased, and TGF-β (Th3 cytokine) increased. They also suggested the presence of an immune imbalance in glaucomatous eyes (Wong et al., 2015). In sum, whether *CPAMD8* is involved in the immunological abnormalities of iris stroma in some subtypes of glaucoma is unknown. In the current study, we confirmed that *CPAMD8* variants co-segregated with PDS/PG in two pedigrees. Further studies are needed to clarify whether *CPAMD8* plays a role in the pathogenesis of iris stromal abnormalities (such as immunological abnormalities), iris epithelial cell loss, and pigment dispersion. In addition, the CPAMD8 protein was mainly detected in the nonpigmented epithelial cells of iris tissues (Siggs et al., 2020; Li X. et al., 2021b), which also implied that the effect of *CPAMD8* variants on the loss of iris pigment epithelium may be indirect.

It is worth mentioning that iris transillumination defects, which are a feature of PDS and PG, were not observed in any patients in the two pedigrees or in sporadic PG patients, even though they presented with other typical clinical phenotypes of PDS/PG, including Krukenberg’s spindle and heavily and homogenously pigmented TM. One of the reasons is probably the fact that iris transillumination defects cannot be detected in darkly pigmented irides of East Asian patients *via* traditional slit-lamp examination (Qing et al., 2009). However, infrared imaging techniques may help demonstrate iris transillumination defects in these patients who show other clinical signs of PDS (Roberts and Wernick, 2007).

In conclusion, our study identified compound heterozygous variants of the *CPAMD8* gene in these two Chinese pedigrees with PG inherited in an autosomal recessive manner. This study reported a possible association between the biallelic *CPAMD8* variants and PDS/PG. Because the *CPAMD8* ortholog is conspicuously absent in rodent genomes (Siggs et al., 2020), animal studies of the pathogenesis of PDS/PG with *CPAMD8* variants require special considerations.

## Data Availability

The data analyzed in this study is subject to the following licenses/restrictions: The datasets for this article are not publicly available due to concerns regarding participant/patient anonymity. Requests to access these datasets should be directed to XL, xliu1213@126.com.
