# Supplementary material for: Compound Heterozygous Variants of the CPAMD8 Gene Co-Segregating in Two Chinese Pedigrees With Pigment Dispersion Syndrome/Pigmentary Glaucoma
Source: Front Genet. 2022 Jul 25;13:845081. doi: 10.3389/fgene.2022.845081 (PMC9358689; doi:10.3389/fgene.2022.845081)
Supplement: Supplementary file 1 [file Table1.docx]

**Table S1. Clinical data of members except fo patients in two PG pedigrees**

| **Pedigree** | **Patient** | **diagnosis** | **Gender** | **Changes of nucleotide and amino acid** | **Age at last exam**  **(years old)** | **BCVA** | | **Last IOP (mmHg)** | | **Krukenberg spindle*** | | **TM pigmentation**** | | **C/D ratio** | | **Visual field** | | **RNFL (μm)** | |
| --- | --- | --- | --- | --- | --- | --- | --- | --- | --- | --- | --- | --- | --- | --- | --- | --- | --- | --- | --- |
|  |  |  |  |  |  | **OD** | **OS** | **OD** | **OS** | **OD** | **OS** | **OD** | **OS** | **OD** | **OS** | **OD** | **OS** | **OD** | **OS** |
| 1 | I:1 | N | M | +/c.1015G>A, p.V339M | 65 | 0.6 | 0.6 | 11 | 10 | 0 | 0 | 1 | 1 | 0.4 | 0.4 | N | N | NA | NA |
|  | I:2 | N | F | c.520C>T, p.R174W/+ | 65 | 1.0 | 0.8 | 17 | 16 | 0 | 0 | 2-3 | 2-3 | 0.3 | 0.3 | N | N | NA | NA |
|  | III:1 | N | M | +/c.1015G>A, p.V339M | 15 | NA | NA | NA | NA | 0 | 0 | 0 | 0 | 0.3 | 0.3 | NA | NA | NA | NA |
|  | III:2 | N | F | c.520C>T, p.R174W/+ | 11 | 0.3 | 0.25 | 15 | 16 | 0 | 0 | 0 | 0 | 0.3 | 0.3 | NA | NA | NA | NA |
|  | III:3 | N | M | +/c.1015G>A, p.V339M | 11 | 0.3 | 0.3 | 14 | 16 | 0 | 0 | 0 | 0 | 0.3 | 0.3 | NA | NA | NA | NA |
|  | III:4 | N | F | c.520C>T, p.R174W/+ | 10 | 0.8 | 1.0 | 15 | 15 | 0 | 0 | 0 | 0 | 0.6 | 0.6 | NA | NA | NA | NA |
|  | III:5 | N | M | +/ c.1015G>A, p.V339M | 10 | 0.8 | 0.8 | 18 | 18 | 0 | 0 | 0 | 0 | 0.2 | 0.2 | NA | NA | NA | NA |
|  | III:6 | N | F | NA | 6 | 0.8 | 0.8 | 15 | 13 | 0 | 0 | 0 | 0 | 0.2 | 0.2 | NA | NA | NA | NA |
| 2 | I:1 | N | M | c.1015G>A, p.V339M; c.1931A>G, p.Y644C/+ | 75 | NA | NA | NA | NA | 0 | 0 | 0 | 0 | NA | NA | NA | NA | NA | NA |
|  | I:2 | N | F | +/c.3238G>A, p.G1080S | 71 | NA | NA | NA | NA | NA | NA | NA | NA | NA | NA | NA | NA | NA | NA |
|  | III:1 | N | M | +/c.3238G>A, p.G1080S | 27 | 1.0 | 1.0 | 16 | 17 | 0 | 0 | 0 | 0 | 0.3 | 0.3 | N | N | 90 | 89 |
|  | III:2 | N | M | NA | 17 | 1.0 | 1.0 | 16 | 16 | 0 | 0 | 0 | 0 | 0.3 | 0.3 | N | N | 95 | 92 |

Abbreviations: BCVA, best corrected visual acuity; C/D, cup-to-disc; F, female; IOP, intraocular pressure; M, male; N, normal; NA, not available; OD, right eye; OS, left eye; OU, both eyes; RNFL, retinal nerve fiber layer; TM, trabecular meshwork.

*Grade of Krukenberg spindle: 0 is defined as none; 1, few flecks; 2, subtle spindle; 3, dense spindle; 4, diffuse pigment (Tandon et al., 2019).

**Grade of TM pigmentation: 0 is defined as no pigment; 1, light pigment, 2, moderate pigment; 3, heavy and non-confluent pigment; 4, heavy and confluent pigment (Tandon et al., 2019).
